# A contemporary analysis of morbidity and outcomes in cytoreduction/hyperthermic intraperitoneal chemoperfusion

**DOI:** 10.1002/cam4.80

**Published:** 2013-04-16

**Authors:** Michelle Haslinger, Valerie Francescutti, Kristopher Attwood, Judith Andrea McCart, Marwan Fakih, John M Kane, Joseph J Skitzki

**Affiliations:** 1 Department of Surgery, University of BuffaloBuffalo, New York; 2 Department of Surgical Oncology, Roswell Park Cancer InstituteBuffalo, New York; 3 Department of Biostatistics and Bioinformatics, Roswell Park Cancer InstituteBuffalo, New York; 4 Department of Surgery, Mount Sinai Hospital and the University of TorontoToronto, Ontario, Canada; 5 Department of Internal Medicine, University of MichiganAnn Arbor, Michigan

**Keywords:** Carcinomatosis, colorectal cancer, cytoreduction, HIPEC, morbidity

## Abstract

The risks and benefits of cytoreductive surgery combined with hyperthermic intraperitoneal chemotherapy (CS/HIPEC) continue to be debated by the oncology community. A retrospective analysis of contemporary data (2003–2011) was performed to provide objective information regarding surgical morbidity, mortality, and survival for patients undergoing CS/HIPEC at a comprehensive cancer center. While procedure-associated morbidity was comparable to other major surgical oncology procedures, there was no operative or 30-day mortality and 60-day mortality was 2.7%. Increasing numbers of bowel resections were found to correlate to an increased incidence of deep surgical site infections (including abscess and enterocutaneous fistula) and need for reoperation which was in turn associated with a decreased overall survival (OS) and progression-free survival (PFS). Five-year OS rates varied by site of tumor origin and histology (disseminated peritoneal adenomucinosis [91.3%], Mesothelioma [80.8%], Appendiceal Adenocarcinoma [38.7%], and Colorectal Adenocarcinoma [38.2%]). With an acceptable morbidity and mortality rate, CS/HIPEC should be included as an effective treatment modality in the multidisciplinary care of select patients with peritoneal metastases.

## Introduction

Historically, peritoneal carcinomatosis (PC) from adenocarcinomas of nongynecologic origin was considered virtually incurable with an average life expectancy of 6 months [1, 2]. Even with the best systemic chemotherapy regimens, current median overall survival (OS) and disease-free survival (DFS) are only 20 and 10 months, respectively [3–5]. The role of surgery for PC has slowly evolved from palliation to potential curative intent. Attempting to remove all visible tumor deposits, “surgical cytoreduction” was first reported in the 1930s for ovarian cancer and eventually became an accepted therapy with proven survival benefit [6–9]. Several decades later, the clinical application of cytoreduction for nongynecologic malignancies and the addition of concurrent hyperthermic intraperitoneal chemotherapy (HIPEC) for the purpose of eliminating remnant microscopic disease was described [10]. More recently, the use of cytoreductive surgery (CS)/HIPEC for nongynecologic malignancies has expanded and is based upon the concept that carcinomatosis is a locoregional phenomenon requiring a locoregional treatment [11].

The risks and benefits of CS/HIPEC for nongynecologic malignancies continue to be vigorously debated in the oncology community despite a growing body of evidence regarding clinical efficacy. Eleven Phase II studies demonstrate a 5-year OS rate ranging from 25% to 47% for colorectal PC treated by cytoreduction and HIPEC, which prompted a Phase III randomized controlled study in this population [12]. The standard therapy group received palliative surgery and “best systemic therapy” consisting of fluorouracil and leucovorin, while the experimental group underwent maximal cytoreduction and HIPEC followed by “best systemic therapy”. A significant benefit in OS favoring CS/HIPEC compared to standard therapy alone, 21.6 versus 12.6 months, was reported. Recently updated data shows a 6-year survival rate of 5% in the standard arm versus 20% in the CS/HIPEC arm [13]. Five-year survival for patients who achieved a complete cytoreduction (CCR0) was an astounding 45%, in contrast to incomplete cytoreduction patients whose median survival was <1 year. Regardless of potential selection bias, these reports collectively suggest that a cohort of carcinomatosis patients truly benefit from this approach and that current systemic chemotherapy regimens are still unlikely to match the observed differences in survival or long-term cures generated by CS/HIPEC [3, 14]. However, morbidity remains a concern as many studies report a 27–56% perioperative complication rate [15]. The Phase III study published in 2003 had an 8% mortality rate with some centers reporting mortality up to 11% [12, 15].

To determine more contemporary rates of morbidity and mortality associated with CS/HIPEC, we have reviewed our institutional experience with this procedure over a recent 8-year period. Our goal is to provide objective data regarding the safety and efficacy of performing cytoreduction and HIPEC in the setting of a comprehensive cancer center.

## Material and Methods

### Patients

All patients who underwent CS/HIPEC at Roswell Park Cancer Institute (RPCI) from 2003–2011 were retrospectively reviewed. Institutional ethics approval for this review was obtained. Strict and uniform inclusion criteria for surgical eligibility included: disease localized to the peritoneal cavity, no distant organ metastases (including the liver or lung), and a baseline functional status of Eastern Cooperative Oncology Group (ECOG) ≤2. In an attempt to define factors associated with the morbidity of the CS/HIPEC procedure, all patients were included for analysis. Patients were evaluated retrospectively from their date of CS/HIPEC at RPCI to an end point consisting of either date of last follow-up or death. Patients that underwent surgical exploration and were not deemed a candidate for CS/HIPEC were excluded from this study, as were patients only undergoing CS without HIPEC.

### Surgery

All CS/HIPEC surgeries were performed by two surgeons, using the same technique. Through a generous midline laparotomy incision, inspection of the abdomen was performed to determine the feasibility of obtaining a CCR. Disease involving the porta hepatis, duodenum, major vascular structures, or an extent of small bowel serosa involvement that would preclude a CCR0 did not undergo CS/HIPEC. Patients who were deemed appropriate for CS underwent greater and lesser omentectomy, resection of the falciform ligament and ligamentum teres, resection of prior abdominal scars, and bilateral salpingo-oophorectomy in female patients was always performed. Additional sites of gross disease were cytoreduced as indicated by visceral resections, peritoneal stripping, and/or fulguration or argon beam coagulation. A CCR0 was classified as those patients who had all visible tumor nodules resected, with no further nodules or implants >2 mm. An incomplete cytoreduction (CCR1 or greater) was considered to include patients that had residual tumor nodules after cytoreduction >2 mm.

Once CS was completed, closed HIPEC was initiated by placing a single outflow cannula within the anterior pelvis and two inflow cannulae at the base of the hemidiaphragms. The cannulae exited through the midline incision, which was temporarily closed with a running suture. Temperature probes were inserted into the cannulae. Three liters of a balanced saline solution were instilled into the peritoneum via the HIPEC circuit, air was evacuated from the circuit, and flow and warming of the fluid commenced. When the inflow and outflow temperatures reached ∼41°, HIPEC was performed for 60–120 min. All patients received 30 mg of Mitomycin C (MMC) via HIPEC. An additional 10 mg MMC was administered at 60 min. The minimal acceptable flow rate was 500 mL/min with no limit on the maximum achievable flow rate. At the end of HIPEC, the chemotherapy was evacuated and the abdominal cavity irrigated with 3 L of crystalloid via the closed perfusion circuit. Upon reopening of the abdominal cavity, the viscera were inspected for any potential injury associated with the HIPEC. Bowel anastomoses were performed at this time. The need for a diverting ostomy was at the discretion of the surgeon. Placement of Seprafilm (Sanofi US, Bridgewater, NJ) in the midline was followed by fascial closure. Prior to skin closure with staples, a closed suction drain was placed in the subcutaneous tissues to address any HIPEC-induced fat necrosis. Chest tubes and total parenteral nutrition (TPN) were not routinely utilized. All patients went to the intensive care unit (ICU) immediately postoperatively.

### Demographics

Baseline patient characteristics were divided into categorical and continuous measures. Categorical measures included gender, primary tumor location, histology, preoperative chemotherapy use, ostomy creation at time of HIPEC, and completeness of cytoreduction. These were reported as frequencies and relative frequencies. Continuous measures included age, postoperative ICU stay (days), total length of hospital stay (days), estimated blood loss, number of bowel resections, and anastomoses with means and standard deviations.

Primary tumor location was classified as colorectal, appendix, or other (small bowel, gastric, ovarian, pancreatic, primary peritoneal). Tumor histology was classified as colorectal adenocarcinoma, appendiceal adenocarcinoma, disseminated peritoneal adenomucinosis (DPAM), mesothelioma, or other (adenocarcinoid, sarcoma, ovarian serous papillary).

### Complications

Complications were recorded as binary variables and reported as frequencies and relative frequencies. These included superficial surgical site infection, deep surgical site infection (anastomotic leak, enterocutaneous fistula [ECF], or abscess), pulmonary (pneumonia, pleural effusion), cardiac (myocardial infarction, arrhythmia), renal (renal failure), urinary tract infection (UTI), venous thromboembolism (deep vein thrombosis, pulmonary embolus, portal vein thrombosis), gastrointestinal (GI; pancreatitis, ascites, ileus, clostridium difficile colitis, TPN requirement), hematologic (anemia, bleeding), neutropenia (absolute neutrophil count <1000), and other (line sepsis, bacteremia, lower extremity compartment syndrome). Given the anticipated length of stay (LOS) we cite for our patients being 14 days or less, LOS >14 days was used as a surrogate for a complicated postoperative course in this cohort even if no defined complication had occurred.

Any association between baseline patient characteristics and incidence of postoperative complication were assessed using logistic regression modeling. Patient characteristic was listed as the independent variable and complication was the dependent variable. From these models, odds ratios were obtained with a 95% confidence interval. A *P*-value of <0.05 indicated a statistically significant relationship.

### Survival

OS and progression-free survival (PFS) were generated using Kaplan–Meier method. Kaplan–Meier curves were also used to show associations with variables and compared with Log Rank tests.

## Results

A total of 112 patients underwent surgical exploration and CS/HIPEC (Table 1). The median patient age was 53 years and the majority of patients were female (59.8%). Median operative time was 12 h and median estimated blood loss was 200 mL. The median number of bowel resections and anastomoses was 1 with the majority of patients (90.2%) avoiding the creation of an ostomy at the time of CS/HIPEC. The median length of ICU and overall hospital stay were 1 and 12 days, respectively. There were no operative or 30-day mortalities and the 60-day mortality rate was 2.7%.

**Table 1:** Characteristics of patients undergoing CS/HIPEC

Demographic data		
Age	Median years (range)	53 (16–79)
Gender	Male (%)	45 (40.2%)
Primary tumor site	Colorectal (%)	38 (33.9%)
	Appendix (%)	51 (45.5%)
	Other^1^(%)	23 (20.5%)
Tumor histology	Colorectal adenocarcinoma (%)	38 (33.9%)
	Appendiceal adenocarcinoma (%)	24 (21.4%)
	DPAM^2^(%)	27 (24.1%)
	Peritoneal mesothelioma (%)	11 (9.8%)
	Other^3^(%)	12 (10.7%)
Preoperative chemotherapy	(%)	51 (45.5%)


Operative data		
Ostomy	(%)	11 (9.8%)
Bowel resections	Median (range)	1 (0–3)
	0	40 (35.7%)
	1	42 (37.5%)
	2	23 (20.5%)
	3	7 (6.3%)
GI anastomoses	Median (range)	1 (0–3)
Operative time	Median hours (range)	12 (6–20)
Estimated blood loss	Median mL (range)	200 (0–2500)
Complete cytoreduction	(%)	72 (64.3%)
HIPEC dose reduction	(%)	29 (25.9%)
Reoperation rate	(%)	11 (9.8%)
ICU stay	Median days (range)	1 (0–21)
Length of hospital stay	Median days (range)	12 (6–122)
Follow-up time	Median months (range)	25 (1–80)

DPAM, disseminated peritoneal adenomucinosis; GI, gastrointestinal; HIPEC, hyperthermic intraperitoneal chemotherapy; ICU, intensive care unit.

1 Includes small bowel, gastric, ovarian, pancreatic, primary peritoneal.

2 Disseminated peritoneal adenomucinosis.

3 Adenocarcinoid, sarcoma, ovarian serous papillary.

The most common histology encountered was adenocarcinoma, with tumors of colorectal origin being the most common indication for CS/HIPEC. Nearly half of the patients received some form of preoperative chemotherapy. The majority of patients (74%) received the anticipated full dose of MMC during HIPEC with dose reduction in the remainder due to extensive preoperative chemotherapy exposure or body habitus as determined by the operating surgeon. A CCR0 was achieved in the majority of patients (64.3%). The 35.7% of patients that did not receive a complete cytoreduction (CCR1 or greater) received HIPEC.

Considering operative morbidity and mortality, superficial surgical site infection was noted most commonly in 15.2% of patients (Table 2). Deep surgical site infections including anastomotic leak, ECF, and abscess occurred in 10.7% of patients. GI complications not attributed to the above mentioned deep surgical site infections occurred in 14.3% of patients. The remainder of complications including cardiac, pulmonary, renal, UTI, venous thromboembolism, hematologic, neutropenia occurred infrequently. Directly related to HIPEC, neutropenia occurred in 6.3% of patients. As a surrogate for morbidity, length of stay in the hospital (LOS) was >14 days for 24.1% of patients. Reoperation, either at the time of admission for CS/HIPEC or within 30 days, was required for 6.3% of patients due to the need for enteric diversion or hemostasis. Median days to reoperation was 11, with six surgeries occurring for anastomotic leak, three for incisional infection/infected mesh, one for bleeding requiring hemostasis, and one for compartment syndrome of the lower extremity requiring fasciotomy related to positioning in stirrups.

**Table 2:** Operative morbidity and mortality

Variable	*n*	%
Morbidity		
Superficial surgical site infection	17	15.2
Deep surgical site infection	12	10.7
Pulmonary complications	4	3.6
Cardiac complications	3	2.7
Renal complications	1	0.9
UTI	5	4.5
Venous thromboembolism	5	4.5
Gastrointestinal complications	16	14.3
Hematologic complications	1	0.9
Neutropenia	7	6.3
Other^1^	4	3.6
Mortality		
30-day mortality	0	0
60-day mortality	3	2.7
Operative mortality	0	0

UTI, urinary tract infection.

1 Includes line sepsis, bacteremia, lower extremity compartment syndrome.

The potential association between baseline patient treatment related factors and complications was assessed using logistic regression modeling (Table 3). Significant associations were observed between superficial surgical site infection and placement of an ostomy (*P* = 0.007). Prolonged ICU stay was associated with a deep surgical site infection (*P* < 0.001), and need for reoperation on the same admission (*P* = 0.009). Overall length of hospital stay was prolonged by a deep surgical site infection (*P* = 0.003) and need for reoperation (*P* = 0.032). Increasing number of bowel resection was associated with a deep surgical site infection (*P* = 0.004) and need for reoperation (*P* = 0.006).

**Table 3:** Factors related to risk of complication following CS/HIPEC

Complication	Dependent variable	Odds ratio (95% CI)	*P*-value
Superficial surgical site infection	Ostomy	6.18 (1.63–23.4)	0.007
Deep surgical site infection	ICU stay	1.54 (1.23–2.02)	<0.001
	LOS	1.16 (1.05–1.27)	0.003
	# of bowel resections	2.79 (1.40–5.56)	0.004
Reoperation	ICU stay	1.35 (1.08–1.70)	0.009
	LOS	1.09 (1.01–1.19)	0.032
	# of bowel resections	3.65 (1.45–9.16)	0.006
	# of anastomoses	4.16 (1.61–10.75)	0.003

ICU, intensive care unit; LOS, length of stay.

**Figure 1:**
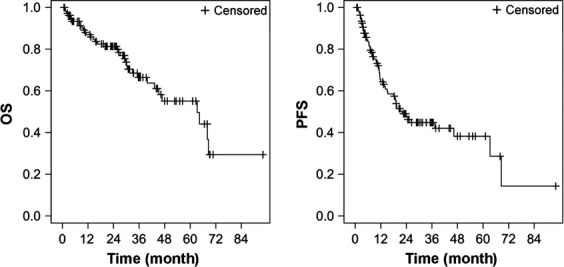
For a mixed population of patients, tumor origin, and tumor histologies, the procedure of cytoreduction and hyperthermic intraperitoneal chemotherapy (HIPEC) was associated with a median overall survival (OS) of 63.2 months with a 5-year survival rate of 55.2% in our series. The median progression-free survival (PFS) was 22 months. Cytoreduction/HIPEC as a procedure did not exhibit any prohibitive or significant procedure-related early mortality in the overall patient population.

**Figure 2:**
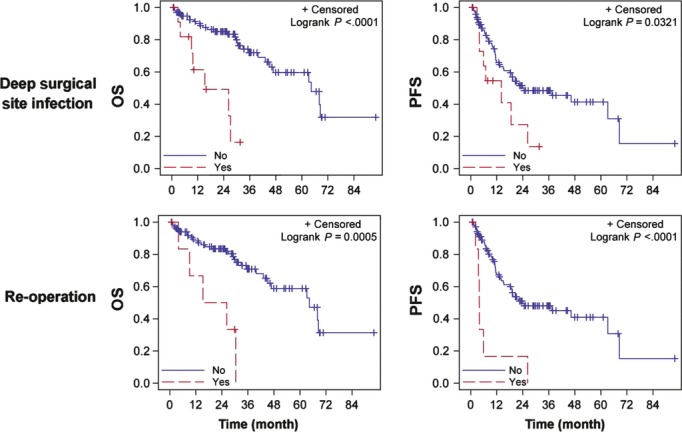
Deep surgical site infection was associated with a reduced 5-year survival from 59.7% of those with no deep surgical site infection down to 16.4%. Progression-free survival was similarly reduced in patients with deep surgical site infection from 41.3% to 13.6% over 5 years. Indicative of a serious complication, patients requiring reoperation had similar reductions of 20.8% and 20.3% in 5-year overall survival and progression-free survival, respectively.

OS and PFS were determined for all patients undergoing the CS/HIPEC procedure (Fig. 1). Of the 112 patients, a total of 35 deaths were observed, with a median survival time of 63.2 months. A total of 55 progression events, including death or recurrence, were observed, with a median progression-free time of 22 months. Complications with significant associations with poor survival were observed between deep surgical site infection and both OS (*P* = 0.0001) and PFS (*P* = 0.0321), and reoperation and both OS (*P* < 0.0005) and PFS (*P* < 0.0001) (Fig. 2).

**Figure 3:**
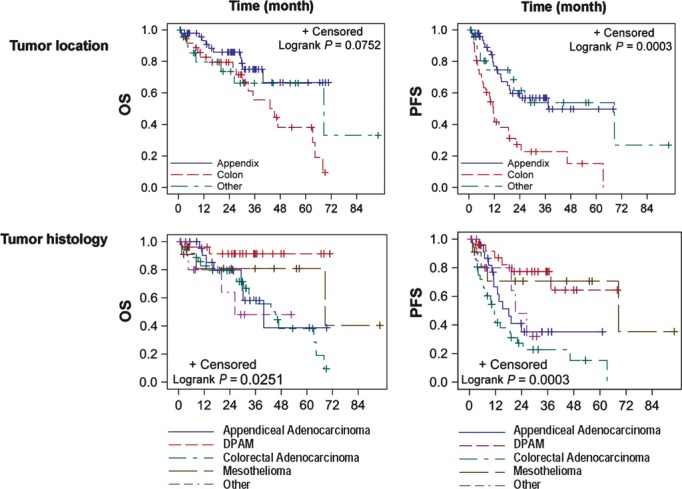
The site of tumor origin had a significant influence on survival. Patients with carcinomatosis from a colorectal primary had a 5-year overall survival of 38.2%, which was significantly lower than appendix origin (66.6%) or other sites (66.2%). The median time to progression exhibited a similar association to tumor location with colon (11.5 months) being less than appendix (37.4 months) or other sites (68.5 months). Tumor histology was also associated with 5-year overall survival as adenocarcinoma of either colon (38.2%) or appendiceal (38.7%) doing worse than disseminated peritoneal adenomucinosis (DPAM) (91.3%), mesothelioma (80.8%), or others (48.0%). A similar pattern was noted for the median time to progression and progression-free survival for these tumor histologies.

Regarding site of tumor origin, colorectal patients had a poorer PFS compared to appendiceal and other sites (*P* < 0.001). Adenocarcinoma histology, regardless of site of origin, was associated with a worse OS (*P* = 0.025) and PFS (*P* < 0.001) compared to other histologies (Fig. 3). The factors associated with OS and PFS and the statistical significance of these associations are summarized in Table 4.

**Table 4:** Survival rates and associated factors following CS/HIPEC

	Five-year OS rate	Median time^1^ (95% CL)	Hazard ratio^2^ (95% CL)	Five-year PFS rate	Median time^1^ (95% CL)	Hazard ratio^2^ (95% CL)
Tumor location						
Colon	38.2%	45.2 (30.4, 64.4)	1.000	15.2%	11.5 (6.5, 19.0)	1.000
Appendix	66.6%	NR (39.9, NR)	0.42 (0.19, 0.91)	49.8%	37.4 (18.1, NR)	0.35 (0.19, 0.63)
Other	66.2%	68.5 (20.0, NR)	0.66 (0.27, 1.59)	53.9%	68.5 (8.2, NR)	0.34 (0.15, 0.75)
Tumor histology						
Colorectal adenocarcinoma	38.2%	45.2 (30.4, 64.4)	1.000	15.2%	11.5 (6.5, 19.0)	1.000
Appendiceal adenocarcinoma	38.7%	39.9 (29.2, NR)	0.78 (0.34, 1.81)	35.2%	18.1 (11.1, NR)	0.58 (0.30, 1.13)
DPAM	91.3%	NR (NR, NR)	0.15 (0.03, 0.64)	64.4%	NR (37.4, NR)	0.19 (0.08, 0.45)
Mesothelioma	80.8%	68.5 (8.2, NR)	0.39 (0.11, 1.36)	70.7%	68.5 (5.4, NR)	0.21 (0.06, 0.70)
Other	48.0%	26.3 (3.8, NR)	1.30 (0.43, 3.89)	32.0%	21.1 (3.3, NR)	0.52 (0.20, 1.35)

OS, overall survival; PFS, progression-free survival; DPAM, disseminated peritoneal adenomucinosis; NR, not reached.

1 Time measured in months.

2 Hazard ratio compared to colon (location) or colorectal adenocarcinoma (histology).

**Figure 4:**
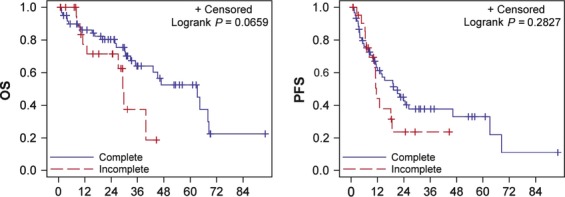
Despite being a heterogeneous population, completeness of cytoreduction in all patients (excluding DPAM) appeared to be associated with improved OS, although this did not reach statistical significance (*P* = 0.0659). PFS was not different in those patients undergoing a complete cytoreduction as compared with an incomplete cytoreduction. DPAM, disseminated peritoneal adenomucinosis; OS, overall survival; PFS, progression-free survival.

Considering complete versus incomplete cytoreduction for all cases excluding DPAM, Figure 4 indicates a trend toward better OS in those patients undergoing a CCR, although this did not reach statistical significance (*P* = 0.0659). While this group is heterogeneous in terms of tumor behavior and prior treatments, the trend toward CCR appeared to be a predictor of survival regardless of histology.

## Discussion

The results of our analyses imply that the surgical procedure of CS/HIPEC in the contemporary setting can be performed safely with minimal postoperative mortality and acceptable morbidity. These data are consistent with low morbidity when including surrogates of complications such as LOS >14 days (24.1% of all patients) and considering the median ICU (1 day) and overall hospital stay (12 days). In this cohort, there was no operative mortality, and the 30- and 60-day mortality was low compared to other major surgical oncology procedures [16–18]. The low morbidity and mortality associated with our series is likely reflective of a strict patient selection, experienced preoperative staff (radiology, pathology, and medical oncology), and postoperative ancillary staff (nursing, respiratory, dietary, and physical therapy). Accordingly, CS/HIPEC has been used at our institution in a multidisciplinary fashion. Nearly half of the patients in our series received preoperative chemotherapy suggesting that CS/HIPEC is viewed as an adjunct, and not a replacement for systemic therapy in select patients. The ability to achieve a CCR0 in 64% of patients is also a reflection of patient selection and a multidisciplinary approach. Performing these often rigorous procedures is enhanced by the resources available to a dedicated comprehensive cancer center and a low mortality and morbidity should be readily achievable in similar settings.

In our series, deep surgical site infection including anastomotic leak, ECF, and abscess, was the most serious morbidity, and this occurred in 10.7% of patients. This appears to be similar to other reported incidences during CS/HIPEC [19–21]. When present, however, our analyses suggest that anastomotic leak/ECF is associated with a lower OS and PFS. These patients often require reoperation for enteric diversion and reoperation itself was associated with a lower OS and PFS. A factor linked to a higher likelihood of deep surgical site infection was the number of bowel anastomoses. Therefore, the need for an increasing number of bowel anastomoses, associated with anastomotic leak/ECF and lower OS and PFS likely reflects aggressive tumor biology and possibly a surrogate for extent of disease, which portends a potentially poorer outcome. While the percentage of patients who received an ostomy at the time of CS/HIPEC was low (9.8%) in this series, it is unclear if deep surgical site infections would have been reduced with diversion.

Inclusive of all tumor origin sites and histologies, the procedure of CS/HIPEC had an excellent OS (median 63.2 months) and PFS (22 months). The site of primary tumor origin appeared to greatly influence survival and is consistent with the previously reported literature [22–25]. Patients whose primary tumor was of colorectal origin had a decreased median PFS as compared with appendiceal or other tumor site origins. Regarding tumor histology, adenocarcinomas of colorectal or appendiceal origin had the lowest survival compared with DPAM and peritoneal mesothelioma. Five-year OS rates for patients treated for adenocarcinomas of colorectal and appendiceal origin were 38.2 and 38.7 months, respectively. Following treatment, the 5-year OS rate for patients with DPAM was 91.3 and 68.5 months for patients with peritoneal mesothelioma. These findings are highly consistent with published data regarding outcomes associated with CS/HIPEC for the various histologies and their associated tumor biology.

While we believe the current analyses demonstrate the utility and safety of CS/HIPEC at our center, there are certain limitations of the study that must be recognized. First, the study is retrospective in nature and any selection bias would be difficult to determine. For example, the patients in this study represent patients who fulfilled our strict criteria for eligibility for CS/HIPEC, which may have improved our survival data compared with older studies. While selection bias remains a possibility, our results support the notion that having strict eligibility criteria may assist in determining the cohort of patients who may achieve the maximal benefits of this procedure, and conversely, those that may not benefit from it. It has been suggested that CS/HIPEC selects the patients that are likely to have a good outcome with any therapy. Our results would strongly argue against this, as almost half of the patients received preoperative chemotherapy and then proceeded to CS/HIPEC for either residual or refractory disease. In the rare patient who clinically and/or radiographically exhibited a complete response to systemic chemotherapy, CS/HIPEC was still performed as these patients often exhibit pathology-proven active disease in the peritoneum found at the time of surgery.

Prognosis and probability of CCR in patients undergoing CS/HIPEC have been shown in a number of studies to be related to the Peritoneal Cancer Index (PCI), a reflection of the extent and location of disease noted in the abdominal cavity upon exploration [26]. Comparing our current study to other CS/HIPEC series is possible when considering the completeness of cytoreduction, but is constrained by our lack of intraoperative PCI scoring. In our evaluated cohort, a large number of patients were pretreated with systemic chemotherapy prior to CS/HIPEC, making the “initial” or true PCI score unknown. In patients who have received multiple lines of systemic chemotherapy and have not exhibited the development of systemic metastases, it is unclear whether PCI scoring at the time of CS/HIPEC would more accurately predict survival or outcome compared to the completeness of cytoreduction.

Another potential limitation for comparison of OS and PFS was the lack of comparison to a similar cohort of patients that received systemic chemotherapy only. Currently, randomized trials comparing CS/HIPEC to systemic chemotherapy are unlikely to accrue a significant number of patients for a variety of reasons as recently demonstrated by ACOSOG Z6091 which was closed for lack of enrollment (one patient accrued in 2 years, with a goal of 340) [27, 28]. While the lack of a control arm may represent a concern for those who view CS/HIPEC as a complete alternative to systemic chemotherapy, we readily acknowledge that CS/HIPEC was employed in a multidisciplinary fashion at our institute, to augment other therapies provided to these patients. Furthermore, preoperative and/or postoperative systemic chemotherapy likely had a major benefit in a significant percentage of our patients and CS/HIPEC should be viewed as a complementary therapy. Lastly, these single institution results may not be able to be extrapolated to other treatment centers. We believe, however, that the utilization of strict eligibility criteria, a strong multidisciplinary approach, and experienced support staff is likely to optimize the outcomes of these patients at similar treatment centers.

In summary, these findings argue against the misconception that the mortality and morbidity associated with CS/HIPEC is prohibitive as has been recently suggested [27, 29–31]. These results also demonstrate that CS/HIPEC may be an effective option in patients with PC from colorectal adenocarcinoma that manifest disease only within the peritoneal cavity, despite receiving systemic chemotherapy. CS/HIPEC has the potential to offer significant survival prolongation in select patients. Patients that require multiple bowel resections and anastomoses during CS/HIPEC may not benefit from this approach, possibly related to tumor biology, and alternative strategies should be explored. Continued investigations into optimal treatment algorithms that include CS/HIPEC are justified.
